# Real-world evidence analysis of palbociclib prescribing patterns for patients with advanced/metastatic breast cancer treated in community oncology practice in the USA one year post approval

**DOI:** 10.1186/s13058-018-0958-2

**Published:** 2018-05-02

**Authors:** J. K. Kish, M. A. Ward, D. Garofalo, H. V. Ahmed, L. McRoy, J. Laney, G. Zanotti, J. Braverman, H. Yu, B. A. Feinberg

**Affiliations:** 1 Cardinal Health Specialty Solutions, 2515 McKinney Ave, Suite 1600, Dallas, TX 75201 USA; 2 Pfizer, Inc., 235 East 42nd Street, New York, NY 10017 USA

**Keywords:** Palbociclib, Real-world, Treatment patterns, Neutropenia, Observational, Breast cancer, Metastatic breast cancer

## Abstract

**Background:**

Rapidly evolving understanding of cancer biology has presented novel opportunities to translate that understanding into clinically relevant therapy. Palbociclib, a novel, first-in-class cyclin-dependent kinase (CDK) 4/6 inhibitor was approved in the USA in February 2015 for the treatment of advanced/metastatic breast cancer. We examined real-world evidence in the first year post approval to understand the clinical and demographic characteristics of patients treated with palbociclib in community oncology practices and the dosing, treatment, and complete blood count (CBC) monitoring patterns.

**Methods:**

This was a retrospective observational study of structured data from a US electronic medical record (EMR) database. Female patients receiving palbociclib after 31 January 2015 were followed through 31 March 2016. Our methodological rules were constructed to aggregate drugs received according to the order in which they are given, i.e., identify the line of therapy as first, second, or third line, etc., using treatment order and course description fields from the EMR.

**Results:**

There were 763 patients initiating palbociclib who met the selection criteria. Of those, 612 (80.2%) received palbociclib concomitantly with letrozole. Mean follow up was 6.4 months and mean age at palbociclib initiation was 64 years. Of patients with a known starting dose (*n* = 417), 79.9% started on palbociclib 125 mg. Dose reductions were observed in 20.1% of patients. Percentages of patients according to line of therapy at initiation of palbociclib were first-line, 39.5%; second-line, 15.7%; third-line, 13.1%; and fourth-line therapy or later, 31.7%. On average, two CBC tests were conducted during the first cycle of palbociclib treatment. Overall, 74.6% of patients had a neutropenic event during follow up including 47.3% and 8.0% of patients with a grade 3 or 4 occurrence, respectively.

**Conclusions:**

Real-world palbociclib use one year post US approval demonstrates a more heterogeneous patient population than that studied in the clinical trials with more than half of the patients receiving palbociclib plus letrozole in later lines of therapy. CBC testing rates suggested good provider compliance with monitoring guidelines in the USA prescribing information. The occurrence of grade 3 and 4 neutropenia (based on laboratory results) was consistent with the rates of grade 3 and 4 neutropenia in two phase-III studies (PALOMA-2, 56% and 10%; PALOMA-3, 55% and 11%, respectively). Understanding palbociclib utilization in real-world patients and how drug dosing and monitoring are performed aids in the understanding of safe and effective use of the drug.

**Electronic supplementary material:**

The online version of this article (10.1186/s13058-018-0958-2) contains supplementary material, which is available to authorized users.

## Background

In 2017 it is estimated that breast cancer accounted for nearly 30% of all new cancers diagnosed in women in the USA and was the leading cause of cancer-related deaths among women aged 20–59 years [1]. Annually approximately 6% of new breast cancer cases present as de novo metastatic disease and as many as 30% of patients diagnosed with early-stage disease have a later recurrence at a distant site (i.e., metastatic disease) over their lifetime [2, 3]. Survival from breast cancer is highly dependent upon the stage at diagnosis; the 5-year relative survival rate from the National Cancer Institute Surveillance, Epidemiology and End Results (NCI-SEER) for women with local or regional disease at diagnosis is 98.8% and 85.2%, respectively, but is only 26.3% for those diagnosed with distant metastatic disease at presentation [2].

Metastatic breast cancer (MBC) remains incurable and presents a significant unmet medical need [4]. Treatment selection for MBC is based on biomarkers including hormone-receptor (HR) and human epidermal growth factor receptor 2 (HER2) status, and individual patient and clinical characteristics that may include tumor burden, timing of disease recurrence, and the type of prior adjuvant therapies. Novel therapies that target the cell cycle and immunotherapies that activate the immune system are resulting in the expansion of the arsenal of systemic cancer treatments for MBC. Many of these treatments are being granted breakthrough therapy designation by the US Food and Drug Administration (FDA) with accelerated approval cycles because of the extreme unmet need. Much progress has been made in the treatment of HER2+ MBC; however, HR+/HER2- disease had not seen any new treatment in a decade prior to the approval of the oral cyclin-dependent kinase (CDK)4/6 inhibitor, palbociclib, in February of 2015. This first-in-class drug warrants evaluation of real-world data on patient characteristics and treatment patterns to aid in the understanding of its safe and effective use in broad clinical practice.

Palbociclib prevents tumor cell proliferation by stopping progression from the G1 to the S phase of the cell division cycle by binding to the RB protein, thereby inhibiting transcription. Palbociclib in combination with the aromatase inhibitor letrozole was granted accelerated approval by the FDA as initial endocrine-based therapy in postmenopausal women with HR-positive/HER2-negative MBC^1^ in February 2015 based on the results of the phase II PALOMA-1 trial [5]. Palbociclib in combination with fulvestrant was approved one year later (February 2016) in premenopausal or postmenopausal women with disease progression following endocrine therapy, based on results from the phase III PALOMA-3 trial [6]. Recently the phase III randomized control trial (RCT), PALOMA-2, confirmed the findings of PALOMA-1, demonstrating median progression-free survival (PFS) of 24.8 months in the arm receiving palbociclib plus letrozole compared to PFS of 14.5 months in the arm receiving placebo plus letrozole (hazard ratio (HR) = 0.58; *P* = < 0.001) [7]. The safety results from all three trials were consistent, with no new safety signals identified in the phase III studies. Subsequently, palbociclib has been granted regular approval in combination with an aromatase inhibitor (AI) for initial endocrine-based treatment [8].

Understanding real-world treatment patterns, patient characteristics and safety outcomes for novel treatments is important to practitioners who may be prescribing a new agent with little or no practical experience. The purpose of this study was to assess the settings in which palbociclib was being initiated (e.g., clinical/demographic characteristics of patients, line of therapy (LOT), i.e., initiation of palbociclib as first-line, second-line, or third-line therapy, etc.) and examine the real-world occurrence of neutropenia and how providers monitored and managed these events (e.g., dose modifications). The results of this research provide insights into these patterns during the first year of adoption of a drug with a novel mechanism of action into clinical practice among community oncologists across the USA.

## Methods

### Study design

This was a retrospective observational cohort study of patients initiating treatment with palbociclib in combination with letrozole in US-based, community oncology practices in the 1-year interval following approval of the drug. Patients were identified from a community-based, multiplatform Navigating Cancer electronic medical record (EMR) database, which includes records dating back to 2007 for more than 2 million patients with cancer, seen by over 975 oncology and hematology providers across more than 50 locations in 25 states. The database contains structured data fields describing diagnoses (ICD-9/10 codes), treatment orders (drug/date), treatment course descriptions (dose/schedule), clinical characteristics (e.g., TNM stage, biomarker testing results, Eastern Cooperative Oncology Group Performance Status (ECOG- PS)), standard laboratory records (e.g., blood chemistry, lipid, renal function tests/panels, etc.), and vital status (through Social Security Death Master File matching and practice input). Review of clinical progress notes or in-depth chart review was not planned or conducted for this study.

Women with at least one treatment record for palbociclib between 1 February 2015 and 31 January 2016, who were at least 18 years of age at initiation of palbociclib, were selected from the database. Patients were excluded from the study cohort if any of the following were true: (1) no breast cancer diagnosis (ICD-9 = 174.x; ICD-10 = C50.x); (2) received palbociclib as part of a clinical trial or a palbociclib prescription prior to 1 February 2015; or (3) no record of combination/concomitant treatment with letrozole or fulvestrant (treatment record within 30 days of palbociclib). Patients receiving palbociclib in combination with fulvestrant were identified but were not included in these analyses as FDA approval of this combination was granted in February 2016 and as such all use during the study time period would be considered off-label. Patients were followed through 31 March 2016 (or until death or loss to follow up).

All patient records were de-identified prior to study cohort selection in accordance with the Health Insurance Portability and Accountability Act (HIPAA) of 1996. A central institutional review board (Western IRB, Puyallup, Washington) reviewed the study protocol and deemed the study exempt from full review. A waiver of informed consent was obtained for the study.

### Line of therapy

To report on the utilization of palbociclib by LOT, methodological rules were developed, which aggregated treatment orders into drug regimen combinations (and hence LOTs) based on the dates of those treatment orders and course descriptions (days of supply) [9]. EMR treatment records are created from the manual entry by the provider or staff of the drug name, dose, and course description into the EMR system. In this database, treatment records are not linked to either the practice’s dispensing pharmacy claim file (regardless if present or not) or to a payer-based administrative claims database. As such, refill orders for oral agents (e.g., letrozole, palbociclib) were not captured unless the provider indicated a new treatment order for the refill.

The first treatment regimen observed following metastatic diagnosis was assigned as MBC LOT 1. The date of metastatic diagnosis was defined based on the date of the earliest of records of any of the following: American Joint Committee on Cancer (AJCC) stage IV, ICD-9/10-CM code for metastatic disease, or M1 stage. If none of the referenced clinical data points were recorded in the EMR, a patient was assigned a metastatic diagnosis date on the date of the first treatment order for palbociclib. Treatment orders occurring within 30 days were aggregated into treatment regimen combinations. Any treatment order for drug beyond 30 days signaled the initiation of a new LOT. An exception to this rule was made for patients receiving letrozole monotherapy (regardless of the duration of letrozole treatment) who then received palbociclib in combination with letrozole. This patient was considered to have received palbociclib + letrozole in a single LOT. Similarly, patients receiving palbociclib in combination with letrozole, with a subsequent order for palbociclib, but no indication of a refill of letrozole, were considered to remain on the combination treatment.

### Treatment cycles

As an active treatment interval for palbociclib + letrozole could not be accurately defined due to the potential for missing prescription refill data for oral therapies (only the interval between initiation and discontinuation is known based on treatment record data), patients were assumed to be receiving palbociclib from date of initiation through the following (examined in order described): date of death (if deceased), date of next LOT – 1 day (if received subsequent LOT), or the date of last follow up (last medical encounter or visit/last treatment order). As such, given that an actual cycle number was not available from the treatment course description, the cycle during which a complete blood count (CBC), neutropenic event or other occurred was based on the days since initiation of treatment, where 56 days equaled two cycles, 112 days equaled 4 cycles, and 168 days equaled 6 cycles.

### Adverse event monitoring

Hematologic toxicities were the only directly measurable grade III/IV adverse event (AE) that represented > 1% in the PALOMA trials. We therefore examined the frequency of CBC based on laboratory records within the structured data fields of the EMR by cycle of palbociclib therapy. An absolute neutrophil count below 2000 cells/mm^3^ was considered evidence of a neutropenic event. Grade of the event was assigned as follows: 1500–1999 cells/mm^3^ = grade 1; 1000–1499 cells/mm^3^ = grade 2; 500–999 cells/mm^3^ = grade 3; and < 500 cells/mm^3^ = grade 4, consistent with common terminology for adverse events (CTCAE) v4.0 criteria.

### Analysis

Only descriptive statistics (e.g., means, medians, proportions) were calculated for outcomes assessed in this study. All analysis was conducted in SAS v9.4 (SAS Institute, Cary, NC, USA). LOT at initiation (first through fourth or later, or first or second or later) was used as the primary stratification variable for analysis. Stratification by length of follow up (<6 months or ≥ =6 months) was also conducted to account for the variable follow up of patients and adjust for any potential information bias. Patients were considered lost to follow up if the patient was not deceased and the time between last medical/treatment record and the end of the study period (1 March 2016) was > 90 days.

## Results

### Study sample

Overall, 965 adult women with a treatment order for palbociclib were identified. Of the initial 965 women, 763 (79.1%) were retained for analysis. The 202 patients removed from analysis were excluded sequentially due to the following reasons: (1) no breast cancer diagnosis, *n* = 13; (2) received palbociclib as part of a clinical trial or a palbociclib prescription prior to 1 February 2015, *n* = 21; and (3) no record of combination treatment with letrozole or fulvestrant, *n* = 168. Of these 763 patients, 80.2% (*n* = 612) were treated with palbociclib + letrozole and for whom data are reported.

### Patient demographics and clinical characteristics

The mean age at initiation of palbociclib was 64 years (SD = 12) with 49.7% (*n* = 304) patients < 65 years of age and 50.3% ≥ 65 years of age including 20.6% of patients ≥ 75 years of age (Table 1). The highest AJCC stage recorded in the EMR was recurrent cancer, 38.4%; for de novo metastatic cancer, 45.4%, and for unknown, 16.2% (restaging of patients is not typically recorded in the EMR). More than two thirds (69.9%) of patients had a confirmed HR+/HER2- tumor; data were not available for the remainder. Site of metastatic disease was not available in the structured EMR database for 95.8% of patients (data not shown).

**Table 1:** Demographic and clinical characteristics of patients initiating palbociclib + letrozole

	All palbociclib + letrozole patients (*N* = 612)	
Age (years)		
At advanced breast cancer diagnosis (mean (SD))	61	(12)
At initiation of palbociclib (mean (SD))	64	(12)
<65 (*n* (%))	304	(49.7)
65–74 (*n* (%))	182	(29.7)
≥75 (*n* (%))	126	(20.6)
Weight (lbs) at initiation of palbociclib (mean (SD))	165	(37)
BMI (mean (SD))	28.2	(7.6)
Histology (*n* (%))		
Invasive ductal	190	(31.0)
Invasive lobular	7	(3.7)
Unknown	415	(67.8)
Stage recorded in EMR^a^ (*n* (%))		
Recurrent	198	(38.4)
De novo stage IV	278	(45.4)
Unknown	99	(16.2)
ECOG at palbociclib treatment initiation (*n* (%))		
0/1	348	(56.9)
2	77	(12.6)
≥3	18	(2.9)
Unknown	169	(27.6)
ER+/HER2 -		
Yes	428	(69.9)
Unknown	184	(30.1)
ER status (*n* (%))		
Positive	505	(82.5)
Negative	33	(5.4)
Unknown	74	(12.1)
PR status (*n* (%))		
Positive	388	(63.4)
Negative	123	(20.1)
Unknown	101	(16.5)
HER2 status (*n* (%))		
Positive	38	(6.2)
Negative	465	(76.0)
Unknown	109	(17.8)

*BMI* body mass index, *EMR* electronic medical record, *ECOG* Eastern Cooperative Oncology Group, *ER* estrogen receptor, *HER2* human epidermal growth factor receptor 2, *PR* progesterone

^a^Stage recorded in the EMR at the closest date to palbociclib initiation; restaging of patients does not typically occur

**Fig. 1:**
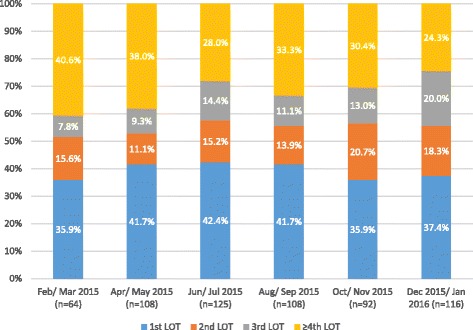
Proportion of patients initiating palbociclib + letrozole as first-line, second-line, third-line or fourth-line or later treatment by month of initiation. LOT, line of therapy

Overall, 39.5% of patients initiated palbociclib + letrozole in LOT 1 while 15.7%, 13.1% and 31.7% initiated palbociclib + letrozole in LOT 2, LOT 3 and LOT 4+, respectively (Additional file 1: Table S1). LOT at initiation of palbociclib in February/March 2015 compared to December 2015/January 2016 ranged from 35.9% to 37.4% of patients for LOT 1 (Fig. 1.) Over this 1-year interval the largest change in the proportion of patients who received palbociclib + letrozole was for LOT 4+, declining from 40.6% in February/March 2015 to 24.3% in December 2015/January 2016. This decrease corresponded to an increase in the proportion of patients receiving palbociclib + letrozole in LOT 3 from 7.8% to 20.0%, from 15.6% to 18.3% in LOT 2 and from 35.9% to 37.4% in LOT 1. The mean length of follow up from initiation of palbociclib + letrozole to the end of the study period was 6.4 months (SD = 3.9) (Additional file 1: Table S1). At the end of follow up, 78.8% (*n* = 482) of patients were on treatment (with 61.4% (*n* = 296) of patients on treatment still receiving palbociclib + letrozole), 9.5% (*n* = 58) were lost to follow up, and 72 (11.8%) were deceased. Of those deceased, 70.8% (*n* = 51) had received a LOT following palbociclib + letrozole while 29.2% (*n* = 21) had not received another LOT. Of the 21 deceased patients who did not receive another LOT, 18 patients initiated palbociclib in LOT 2+ and had < 6 months of follow up.

There were 417 of patients (68.1%) with a known palbociclib starting dose (Table 2). Of the 417 patients with a known starting dose, 88.0% (*n* = 367) initiated palbociclib at 125 mg, 11.0% at 100 mg, and 1.0% at 75 mg. Dose was reduced in 20.1% (*n* = 84) patients with known starting dose. All dose reductions (*n* = 84) occurred within 168 days of palbociclib treatment initiation (approximately first 6 cycles assuming 28 days per cycle) while 69.0% (58/84) occurred within the first 2 cycles. Of those patients who did have the initial dose reduced, 77.4% (*n* = 65) had the dose reduced from 125 to 100 mg. The average number of days to the first dose reduction was 48 (SD = 31). The median days to first dose reduction was 39 (data not shown). Of note, five patients had a record of a dose increase, with three dose increases occurring among patients initiating at either 75 or 100 mg and two dose increases occurred among patients who were initially dosed at 125 mg, and had the dose reduced and then subsequently increased.

**Table 2:** Initiation dose of palbociclib and patterns of dose reductions among patients treated with palbociclib + letrozole

	Overall		Number of palbociclib cycles received^a^					
			6 cycles^b^		4 cycles^c^		2 cycles^d^	
Total patients (*n* (%)	612	(100)	336	(54.9)	445	(72.7)	524	(85.6)
Patients with known starting dose	417	(68.1)	269	(80.1)	323	(72.6)	367	(70.0)
Patients with unknown starting dose	195	(31.9)	67	(19.9)	122	(27.4)	157	(30.0)
Starting dose (*n* (% of patients with known starting dose))								
125 mg	367	(88.0)	237	(88.1)	283	(87.6)	321	(87.5)
100 mg	46	(11.0)	30	(11.2)	38	(11.8)	42	(11.4)
75 mg	4	(1.0)	2	(0.7)	2	(0.6)	4	(1.1)
Number of dose reductions (*n* (% of patients with known dose))								
None	333	(79.9)	185	(68.8)	240	(74.3)	309	(84.2)
≥ =1	84	(20.1)	84	(31.2)	83	(25.7)	58	(15.8)
Type of first dose reduction (*n* (% of patients with known dose))								
Reduction from 125 mg to 100 mg	65	(15.6)	65	(24.2)	64	(19.8)	45	(12.3)
Reduction from 100 mg to 75 mg	6	(1.4)	6	(2.2)	6	(1.9)	5	(1.4)
Reduction from 125 mg to 75 mg	13	(3.1)	13	(4.8)	13	(4.0)	8	(2.2)
Days to first dose reduction (mean, SD)	48	(31)	48	(31)	48	(31)	46	(31)

^a^Number of cycles approximated by time since initiation of treatment of palbociclib as cycle/refill dates not available in the electronic medical record

^**b**^Interval from initiation of palbociclib + letrozole through 168 days post initiation among those considered on palbociclib + letrozole at day 168 post initiation

^**c**^Interval from initiation of palbociclib + letrozole through 112 days post initiation among those considered on palbociclib + letrozole at day 112 post initiation

^**d**^Interval from initiation of palbociclib + letrozole through 56 days post initiation among those considered on palbociclib + letrozole at day 56 post initiation

### CBC monitoring and neutropenia

Overall, 57.4% of patients (*n* = 351) had a record of at least one CBC test during palbociclib + letrozole treatment (Table 3). Regardless of cycles completed, the mean number of CBC tests among all patients was 6.0 (SD = 5.7). The mean number of CBC tests during the first cycle (28 days of treatment) was 2.0 (SD = 1.1, data not shown). During the first two cycles (56 days) of treatment the mean number of tests was 3.3 (SD = 2.2), during the first four cycles (112 days) it was 4.7 (SD = 3.6), and during the first six cycles (168 days) it was 5.3 (SD = 4.5).

**Table 3:** CBC laboratory evaluations during palbociclib + letrozole treatment and occurrence, timing and grade of neutropenia

	Throughout palbociclib treatment		First 6 cycles^c^		First 4 cycles^d^		First 2 cycles^e^	
Total patients (*n* (%))	612	(100.0)	497	(81.2)	551	(90.0)	575	(94.0)
Patients with ≥ 1 CBC/WBC during time period (*n* (%))	351	(57.4)	299	(60.2)	316	(57.4)	316	(55.0)
Number of CBC/WBC tests (mean (SD))^a^	6.0	(5.7)	5.3	(4.5)	4.7	(3.6)	3.3	(2.2)
Laboratory value consistent with neutropenia^b^ while on PAL (*n* (%))	262	(74.6)	262	(91.0)	262	(85.3)	243	(76.9)
Days to neutropenia diagnosis (mean (SD))	28.4	(19.6)	28.4	(19.6)	28.4	(19.6)	24.2	(12.6)

*CBC* complete blood count, *WBC* white blood cell count, *ANC* absolute neutrophil count, *PAL* palbociclib

^a^Only patients with at least one laboratory test result were counted in calculating the frequency of CBC/WBC tests, neutrophil count during palbociclib treatment, and grade of neutropenia

^b^Neutropenia = neutrophil percentage divided by 100, multiplied by WBC quantity (neutrophil value in K/μl), and multiplied by 1000 to convert to mm^3^

^**c**^Interval from initiation of palbociclib + letrozole through 168 days post initiation among those considered on palbociclib + letrozole at day 168 post initiation and those who received test/experienced event prior to the end of the interval

^**d**^Interval from initiation of palbociclib + letrozole through 112 days post initiation among those considered on palbociclib + letrozole at day 112 post initiation, and those who received test/experienced event prior to the end of the interval

^**e**^Interval from initiation of palbociclib + letrozole through 56 days post initiation among those considered on palbociclib + letrozole at day 56 post initiation, and those who received test/experienced event prior to the end of the interval

**Fig. 2:**
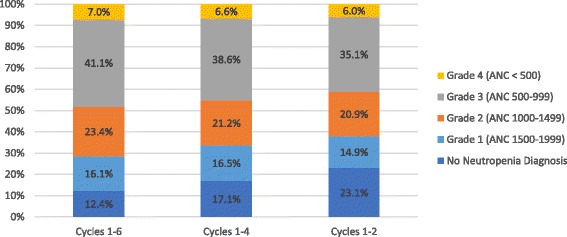
Highest grade of neutropenia diagnosis (%) by number of cycles completed^†^ among patients receiving treatment with palbociclib + letrozole. ^†^Cycles approximated by time since initiation of palbociclib: cycles 1–2 = 1–56 days; cycles 1–4 = 1–112 days; cycles 1–6 = 1–168 days. ANC, absolute neutrophil count

Among the 351 patients with at least one CBC test during palbociclib + letrozole treatment, 74.6% (*n* = 262) had any diagnosis of neutropenia (grade 1 or higher) at a mean 28.4 days (SD = 19.6) following treatment initiation. The median days to first neutropenia diagnosis was 26. The highest grade of neutropenia (consistent with observed laboratory values) anytime during the palbociclib + letrozole treatment by time since initiation of palbociclib (number of cycles) is shown in Fig. 2. Overall, the proportion of patients with laboratory values consistent with grade 3 and grade 4 neutropenia by the 2nd cycle was 35.1% and 6.0%, respectively. By the 4th cycle, the rate of laboratory values consistent with grade 3 and 4 neutropenia increased to 38.6% and 6.6%, respectively. Finally, by the 6th cycle the rate of laboratory values consistent with grade 3 and 4 neutropenia was 41.1% and 7.0%, respectively. By the 6th cycle, 12.4% of patients did not have laboratory values consistent with neutropenia and there had been 123/124 laboratory values consistent with grade 3 and 21/21 laboratory values consistent with grade 4 neutropenia.

## Discussion

This research is the first to examine patient characteristics, dosing and treatment patterns and neutropenia among female patients with MBC treated with palbociclib in combination with letrozole. Often patients treated in practice will be clinically distinct from patients included in RCTs. In this study patients selected from the EMR database appear to have been more heterogeneous compared to the clinical trial population, which is not uncommon in real-world settings. The real-world cohort of patients receiving palbociclib + letrozole were older compared to patients receiving palbociclib in the phase III PALOMA-2 trial (50.3% versus 40.8% ≥ 65 years of age, respectively) and had a higher ECOG-PS score (22.1% among those with known ECOG-PS versus no patients with ECOG ≥2 in the RCT) [7]. In this study we observed use of palbociclib as a later line of therapy in the initial months post approval, which progressively tapered during the ensuing months to the lowest proportion nearly 1 year post approval. In terms of prior treatments, 68.5% of patients had therapy prior to palbociclib + letrozole compared to 62.8% in the PALOMA-2 study [7]. Observing the use of palbociclib in real-world clinical practice is critical to informing clinical decision making and can help providers develop a standard of care for the patient population of interest.

Approximately 88% of patients initiating palbociclib + letrozole started palbociclib 125 mg per recommended prescribing information [8]. We observed dose reductions of palbociclib in 20.1% of patients treated with palbociclib + letrozole. All dose reductions occurred within 6 cycles (168 days) of palbociclib treatment initiation and 66% of those happened within the first two cycles as estimated by EMR entries of treatment, consistent with patterns observed in PALOMA-2 (median days to first dose reduction = 90, range = 28–785, see Finn et al. Supplementary Appendix) [7]. In comparison to dose reductions reported in the clinical trial (36.0% dose reduction due to an AE; initiation at 125 mg), we observed a lower rate of patients (21.2%) who initiated palbociclib at 125 mg and had the dose reduced; this is partially explained by the 11% of patients who initiated treatment at doses lower than 125 mg, which was not allowed in the PALOMA-2 clinical trial [7].

Dose reductions due to adverse events in the clinical trial populations were most often a result of neutropenia. We analyzed neutropenia monitoring in this cohort to assess whether the pattern appeared consistent with the labeled recommendations, that is monitor CBC prior to the start of palbociclib therapy and at the beginning of each cycle and on day 14 of the first two cycles. The mean number of CBC tests conducted during the first cycle was two suggesting provider adherence to labeled recommendations. Among patients who had received at least six cycles of therapy, the rate of grade 3 and 4 neutropenia by laboratory value was 41.1% and 7.0%, respectively, consistent with the rates of grade 3 and 4 neutropenia reported for the clinical trials (PALOMA-1, 57% and 5%; PALOMA-2, 56% and 10%; PALOMA-3, 55% and 11%, respectively). In this analysis, the median days to any neutropenia diagnosis was 26, which is longer than that reported in PALOMA-1 (median time from first dose to first episode of any grade neutropenia in the palbociclib + letrozole group was 20 days); however, we lack precise information on the timing of treatment initiation by the patient unlike the clinical trial data [10]. In the absence of prescription data and start-date correlation, the exact timing of CBC monitoring within the treatment cycle is also an estimate. The slightly higher rate of grade 4 neutropenia may reflect the use of palbociclib as a later line of therapy or it might be related to a more heterogeneous population than was studied in this data set. We noted no difference in the rate of neutropenia in patients who had known prior laboratory values consistent with neutropenia at any time prior (any test following initiation of any treatment) to initiation of palbociclib combination treatment.

### Limitations

The main limitations of this research include potential for incomplete care history, since the breast cancer diagnosis and information for this study was obtained from structured EMR data only. Additionally, we were unable to assess the clinical benefit of palbociclib, given that sufficient time had not elapsed to reliably estimate PFS or overall survival. Care received prior to palbociclib treatment at a practice contributing data to the aggregated EMR database is not included in the structured data fields; therefore, palbociclib + letrozole LOT at initiation may have been misclassified should patients have received treatment prior to or outside of the practices contributing data to the EMR database. In addition, due to the absence of access to chart-level clinical progress notes, it was not possible to verify items such as menopausal status, additional AEs, pathology reports, or laboratory findings of which the interpretation is not available through the structured data fields. These limitations are not unique to this dataset. All EMR studies of this type are also limited by the extent to which data are populated in structured fields, and the degree to which data, such as prescription orders and fills or laboratory results are passively updated within the patient’s record. As this study used EMR data and not pharmacy or claims data, confirmation of palbociclib dispensing (including dose and quantity), other oral drugs used in the course of treatment, or the impact of neutropenia on dose or schedule modifications or clinical complication were not observable. As such, whether a patient received growth factors, had a visit to the emergency department to resolve the neutropenia, or an inpatient admission related to the neutropenia could not be determined.

## Conclusions

Palbociclib has been prescribed to 80,000 patients in the USA since initial approval in February 2015 for the treatment of metastatic breast cancer. Understanding how this first-in-class CDK 4/6 inhibitor is used in a real-world patient population, and how drug dosing and monitoring are performed, aids in the understanding of safe and effective use of the drug. This study demonstrated significant adherence to the recommended starting dose and monitoring patterns for palbociclib in a real-world population that is more heterogeneous with use as a later line of therapy than was studied in PALOMA-1 or PALOMA-2. Neutropenia incidence and dose adjustment patterns were consistent with the PALOMA trials.

## Additional file

Additional file 1: **Table S1.** Line of therapy at palbociclib + letrozole initiation and patient disposition at end of study period. (DOCX 16 kb)

## Footnotes

1 The FDA approved label for palbociclib is for the treatment of HR-positive disease; however, the PALOMA-1 trial specified that patients were estrogen receptor (ER)-positive. We adopt the HR convention for the remainder of the manuscript when referring to either the prescribing information or trial results.
