# Supplementary material for: Real-world evidence analysis of palbociclib prescribing patterns for patients with advanced/metastatic breast cancer treated in community oncology practice in the USA one year post approval
Source: Breast Cancer Res. 2018 May 2;20:37. doi: 10.1186/s13058-018-0958-2 (PMC5932819; doi:10.1186/s13058-018-0958-2)
Supplement: Supplementary file 1 — Table S1. Line of therapy at palbociclib + letrozole initiation and patient disposition at end of study period. (DOCX 16 kb) [file 13058_2018_958_MOESM1_ESM.docx]

## Table S1. Line of therapy at palbociclib + letrozole initiation and patient disposition at end of study period.

|  | **N = 612** | |
| --- | --- | --- |
|  |  |  |
|  |  |  |
|  | N | % |
| Line of therapy at initiation of palbociclib + letrozole |  |  |
| LOT 1 | 242 | (39.5) |
| LOT 2 | 96 | (15.7) |
| LOT 3 | 80 | (13.1) |
| LOT 4+ | 194 | (31.7) |
| Mean follow-up (months) from initiation of palbociclib + letrozole (SD) | 6.4 | (3.9) |
| LOT 1, <6 months of follow-up | 3.0 | (2.0) |
| LOT 1, ≥6 months of follow-up | 9.7 | (2.3) |
| LOT 2+, <6 months of follow-up | 3.0 | (1.7) |
| LOT 2+, ≥6 months of follow-up | 9.6 | (2.3) |
| On treatment at end of study period | 482 | (78.8) |
| Last recorded line of therapy palbociclib + letrozole | 296 | (48.4) |
| Patient received line of therapy following palbociclib + letrozole | 186 | (30.4) |
| Lost to follow-up^¥^ | 58 | (9.5) |
| Last recorded line of therapy palbociclib + letrozole | 35 | (5.7) |
| Patient received line of therapy following palbociclib + letrozole | 23 | (3.8) |
| Deceased | 72 | (11.8) |
| Last recorded line of therapy palbociclib + letrozole | 21 | (3.4) |
| Patient received line of therapy following palbociclib + letrozole | 51 | (8.3) |

^¥^ No treatment/medical encounters within 90 days of end of study period.
